# Controversies in the Management of Papillary Thyroid Cancer Revisited

**DOI:** 10.5402/2011/303128

**Published:** 2011-09-16

**Authors:** Marlon A. Guerrero, Orlo H. Clark

**Affiliations:** ^1^Department of Surgery, University of Arizona, 1501 N. Campbell Avenue, P.O. Box 245131, Tucson, AZ 85724, USA; ^2^Department of Surgery, University of California, San Francisco, CA 94143, USA

## Abstract

The debate on the appropriate treatment of patients with papillary thyroid cancer (PTC) has persisted for several decades. The main controversies focus on the extent of surgery, the timing of central neck dissection, and the indications for radioactive iodine ablation. These controversies continue, for the most part, due to the good prognosis of PTC patients and the questionable effect these treatment modalities have on patient survival. This paper addresses these three controversies and the role of molecular tumor markers in the appropriate treatment selection.

## 1. Introduction

Papillary thyroid cancer (PTC) is both the most common thyroid malignancy and is also the least aggressive thyroid cancer. For this reason, debate has centered on the appropriate extent of treatment, including the extent of surgery (lobectomy versus total thyroidectomy), timing of central neck dissection (prophylactic versus therapeutic), and indications for radioactive iodine ablation and TSH suppressive therapy. There has been a shift in treatment paradigm, as outlined by the most recent American Thyroid Association (ATA) management guidelines, to a risk-stratified, selective management approach [1]. The risk-stratification is based on the risk of recurrence: (1) low risk-completely resected, well-encapsulated tumor, without extrathyroidal extension, without local or distant metastases, absence of aggressive histology, and absence of vascular invasion, (2) intermediate risk-microscopic extrathyroidal invasion, cervical lymph node metastases, ^131^I uptake outside the thyroid bed, and aggressive histology (tall cell, insular, columnar cell carcinoma), or vascular invasion, and (3) high-risk macroscopic tumor invasion, incomplete tumor resection, and distant metastases [1]. These guidelines serve as a guide for the appropriate management of thyroid cancer patients. Yet, despite the plethora of studies on PTC controversy still exists regarding the appropriate extent of treatment. This paper focuses on the three most debated areas in the management of PTC and addresses the utility of molecular markers in operative decision making.

## 2. Extent of Surgery

The extent of disease affects outcomes for PTC. It is uniformly established that patients with PTC and high-risk features and lymph node metastases have an increased risk of recurrence and mortality and substantiates total or near-total thyroidectomy when it can be done safely [2]. This approach also benefits patients with bilateral tumors, tumors with obvious local invasion, and tumors with distant metastases [3, 4]. Furthermore, total thyroidectomy reduced the risk of persistent or recurrent disease, facilitated postoperative ^131^I ablation and whole body scanning, and allows for more sensitive postoperative thyroglobulin monitoring. On the other hand, a minority of experts argue that nearly 80% of patients with PTC can be cured with a thyroid lobectomy and isthmusectomy [5]. Lobectomy eliminates the need for lifelong thyroid hormone replacement and reduces the risk of complications (permanent hypoparathyroidism and recurrent laryngeal nerve injury) [6]. 

Conflicting data exists in the literature on the optimal surgical approach given that there are no prospective, randomized studies addressing the issue. Retrospective data suggest that local recurrence and disease-specific mortality are reduced with a total thyroidectomy compared to subtotal resections [2, 7, 8]. It has been reported that up to 10% of patients treated with lobectomy alone have recurrence in the contralateral lobe and that near-total and total thyroidectomy decrease the risk of recurrent disease [9]. There is also a risk of leaving residual cancer in the contralateral lobe, since more than 50% of patients with PTC have bilateral multifocal disease. Furthermore, even low-risk patients with PTC treated with a lobectomy have a 19% risk of developing nodal metastases at 20 years, compared to 6% for those treated with total or near-total thyroidectomy [9]. Other studies, however, have shown that recurrence and survival is not affected by the extent of surgery in low-risk patients [5, 10] or even in high-risk patients [11]. 

The differences in outcome data reflects the selection bias inherit in retrospective studies. The limitation of selection bias is exemplified in a recent study that concluded that a lobectomy and total thyroidectomy resulted in equivalent survival [12]. However, the perplexing aspect of the study was the finding that the near-total thyroidectomy group had an inferior survival compared to the total thyroidectomy group [12]. The authors' attribute this difference to good patient selection in the use of lobectomy. As a result, the authors' support a lobectomy in patients with low-risk features. Low-risk patients were defined as those with unilateral tumors, tumors < 4 cm, and no evidence of extrathyroidal extension, cervical lymphadenopathy, or distant metastases [12]. However, others have shown that lobectomy alone was associated with a 2.5-fold risk or recurrence and 2.2-fold risk of death compared to those who underwent near-total or total thyroidectomy [13].

Although size is utilized as one criterion for lobectomy, a comprehensive population-based study of over 52,000 patients demonstrated that tumor size affects outcome in patients with PTC [2]. Size was found to directly affect recurrence; the 10-year recurrence rate was 4.6% in tumors < 1 cm, 7.1% in 1.0–1.9 cm, 8.6% in 2.0–2.9 cm, 11.6% in 3.0–3.9 cm, 17.2% in 4.0–7.9 cm, and 24.8% in tumors > 8.0 cm [2]. The extent of thyroidectomy also affected the recurrence rate and overall survival. The 10-year recurrence was 7.7% for patients treated with total thyroidectomy compared to 9.8% for those who underwent lobectomy (*P* < 0.05). In addition, the authors' reported that patients with PTC ≥ 1.0 cm who were treated with a lobectomy had a 15% higher risk of recurrence and a 31% higher risk of death compared to those treated with total thyroidectomy. In subset analysis, this statistical difference was also apparent for tumors 1.0 to 2.0 cm (24% higher risk of recurrence and 49% higher risk of death) [2]. However, there was no difference in recurrence rates and survival for patients with tumors < 1 cm treated with either lobectomy or total thyroidectomy. This study concluded that due to the improved clinical outcomes a total thyroidectomy should be performed for PTC ≥ 1.0 cm. Based upon this study and others, the American Thyroid Association guidelines committee recommended near-total or total thyroidectomy for patients with PTC larger than 1 cm [1].

A recent study, utilizing the Surveillance, Epidemiology, and End Results program database of the National Cancer Institute, questions the validity of the most recent ATA guidelines [14]. The most pressing criticism these authors' had of the study conducted by Bilimoria et al. [2] was the omitted data on disease-specific survival. In this study, nearly 23,000 patients with PTC were analyzed with a mean follow-up of 9.1 years. Mendelsohn et al. [14] confirmed findings from prior reports that demonstrate lower disease-specific survival with increasing tumor size, extrathyroidal extension, advanced age, and positive lymph node status. Interestingly, this study showed that on multivariate analysis, there was no difference in overall- or disease-specific survival between patients treated with lobectomy or thyroidectomy. This lack of survival benefit based on the type of procedure performed was also apparent during subset analysis to account for tumors ≥ 1 cm [14]. The debate regarding the extent of thyroidectomy continues, but we and others suggest that total thyroidectomy enables patients to be followed for recurrence by testing blood thyroglobulin levels and allows for the use of ^131^I screening and ablation. Although a prospective randomized trial would provide guidance, it will probably never be done because of the large number of patients that would be required [15]. The poignant reality unfortunately is that this issue will likely never be completely resolved.

## 3. Prophylactic versus Therapeutic Central Neck Dissection

Currently, the most widely debated subject in patients with PTC is whether a prophylactic central neck dissection (PCND) should be done in patients who are clinically node negative by ultrasound, on physical exam, and by intraoperative assessment. Prophylactic neck dissection is defined as the removal of seemingly normal lymph nodes and fibrofatty tissue during the initial operation. The rationale to perform PCND is the questionable ability to adequately evaluate the central neck compartment with preoperative ultrasonography or with intraoperative assessment [16, 17]. However, others counter that intraoperative surgeon assessment is an accurate predictor of who will benefit from PCND and report only a 3% local recurrence using a selective approach [16]. Occult lymph nodes are common (≥40%) even in patients with small PTC (<1.5 cm) [18]. However, the vast majority of metastases involve only subclinical (<2 mm) microscopic metastases. A French study recently reported that lymph node metastases are common even in patients with small PTC (<2 cm, T1) with no suspicious lymphadenopathy on preoperative ultrasound. The authors' reported that 45% of central and 47% of lateral lymph nodes contain occult metastatic PTC when a PCND was performed [19]. In other reports up to 60%–90% of PTC patients have microscopic or macroscopic lymph node metastases at presentation [20, 21]. 

The issue that remains to be addressed is whether the presence of microscopic lymph node metastases has any adverse effect on clinical outcome. It has been suggested that regional nodal metastases increase the risk of disease recurrence but have minimal to no impact on survival [16, 22]. Other reports document that patients with lymph node metastases experience a higher morality than patients without lymph node involvement [23]. This is also true in patients over 45 years of age with PTC and lymph node metastases [24]. Others, however, have shown no difference in the recurrence rate or disease-specific mortality rate when a total thyroidectomy was performed with or without a PCND [25]. Nonetheless, other benefits have been reported when a PCND is performed. Some contest that PCND provides more accurate staging, specifically in patients > 45 years of age [22, 26]. Another study demonstrated that PCND resulted in lower postablation thyroglobulin levels and more undetectable thyroglobulin levels than those treated with thyroidectomy alone [27]. Similar to other aforementioned studies, this study found no statistical difference in the recurrence rate and mortality between the two groups [27]. These opposing findings in retrospective studies perpetuate the debate rather than provide a uniform approach in the management of PTC. A significant limitation is that studies in support of, or in opposition to, PCND are retrospective and cannot accurately address clinical outcome. A prospective multicenter clinical trial has been submitted to the National Institute of Health regarding the merits and risks of an ipsilateral PCND. 

Several reports have demonstrated that performing a routine PCND during total thyroidectomy is associated with a higher morbidity than thyroidectomy alone [28]. It has been reported that permanent hypoparathyroidism results in about 1%-2% of patients following a total thyroidectomy but can occur in up to 14% of patients with a PCND [29]. Mazzaferri et al. reviewed studies addressing the two approaches and found that there was a higher risk of temporary hypoparathyroidism with PCND compared to thyroidectomy alone [17]. The authors also reported a significantly higher rate of hypoparathyroidism in patients undergoing bilateral PCND compared to unilateral PCND, with no difference in thyroglobulin levels, recurrence or mortality [17]. Other authors' have also corroborated these findings [22]. On the other hand, there was conflicting data for permanent hypoparathyroidism and transient and permanent recurrent laryngeal nerve injury [17]. The higher risk of complications with no difference in outcome in patients treated with PTCN suggests a more temperate approach. As such, the ATA guidelines consider a PCND in patients with T3 and T4 tumors but not for smaller tumors [1]. Others further recommend an approach based on clinicopathological features rather than treating all patients uniformly [30].

## 4. Radioactive Iodine Ablation

Controversy regarding the need for postoperative radioactive iodine (RAI) ablation is on par with the debate on the extent of surgery required to adequately treat PTC patients. RAI has routinely been utilized as an adjunct to surgical therapy for patients with high-risk prognostic factors. The goal of RAI therapy is to eradicate occult persistent or metastatic disease to reduce the risk of recurrence and disease-specific mortality. Given that PTC patients for the most part have an excellent prognosis, the routine use of RAI has been questioned [9]. The most recent ATA guidelines recommend RAI in patients with gross extrathyroidal extension, known distant metastases, and tumors > 4 cm [1]. For patients who do not meet these criteria and have tumors 1 to 4 cm, a selective approach is recommended. RAI is not recommended for tumors < 1 cm, including multifocal micro-PTC, without any high-risk features [1]. Some advocate no RAI ablation for well-differentiated PTC between 1 and 4 cm with less than 3–5 metastatic cervical lymph nodes that are less than 5 mm in diameter [31]. Radioactive iodine ablation is usually recommended for patients with more aggressive histological variants of PTC and for those with primary tumors larger than 2 cm [31].

A recent study from the Memorial Sloan-Kettering Cancer Center revisited the issue of RAI ablation [32]. This retrospective study analyzed 289 patients of which 74% were low-risk and 26% were intermediate-risk according to the ATA risk stratification. Patients were treated with total thyroidectomy (75%) or lobectomy (25%) and selective central neck lymph node dissection (5%). Only 2% of those treated with total thyroidectomy and 4% with lobectomy without RAI recurred. When only tumors > 1 cm were analyzed, the recurrence rate following total thyroidectomy without RAI remained low at 4%. The authors' identified tumor size > 2 cm, lymph node metastases, and ATA intermediate-risk classification as predictors of recurrence. The authors' conclude that selected use of RAI affords good clinical outcome in appropriately selected patients [32]. Previous studies by Mazzaferri and Jhiang [8], reported decreased recurrence in patients treated with ^131^I ablation and with TSH suppression therapy.

Based on current data, the benefits of RAI ablation to prevent persistent or recurrent disease and decrease mortality is not completely clear. The current strategy of selective use affords the best management option and more data regarding the management of such patients is needed.

## 5. Molecular Markers

The controversy concerning the optimal treatment of patients with PTC will continue, especially without data from a randomized, prospective clinical trial. Therefore, other factors will become an essential component in operative management. One of the most promising advances in medicine is the application of personalized genomic medicine. Our understanding of the genetic basis of PTC is evolving and continues to improve. The identification of molecular markers in PTC should help appropriately risk-stratify patients preoperatively by analysis of FNA biopsy specimens and possibly allow for individualized operative planning. The decision regarding the extent of thyroidectomy and node dissection will, therefore, in the future probably rely on preoperative molecular analysis of the tumor.

Cellular signaling pathways have been established as important factors in tumorigenesis. Activation of the mitogen-activated protein kinase (MAPK) signaling pathway is involved in the development of PTC. Several alterations in the signaling pathway have been identified in PTC. One of the most common mutations in PTC involves the BRAF oncogene, occurring in approximately 40% of PTC [33]. BRAF is located on chromosome 7q24 and encodes a serine-threonine kinase that mediates cellular response to growth and differentiation signals. A point mutation in BRAF at codon 600 leads to a valine to glutamate (V600E) alteration and constitutive MAPK pathway stimulation [34]. Nearly 95% of BRAF mutations involve V600E [35]. Other signaling alterations found in PTC include RET/PTC rearrangements (20%–40%), neurotrophic receptor-tyrosine kinase 1 mutations (5%–13%), and RAS proto-oncogene mutations [33]. 

Several studies have associated BRAF mutation with poor prognostic factors. These include advanced age, male gender, extrathyroidal extension, lymph node metastases, and distant metastases [36–39]. BRAF mutation has also been associated with advanced stage and higher risk of recurrent and persistent disease [36, 38]. Other studies have also demonstrated that the BRAF mutation was associated with a lower survival [39]. The association between these high-risk factors makes BRAF the most valuable prognostic indicator to date. Although considerable information has been reported regarding the influence of BRAF mutation as a prognostic indictor of a worse outcome, no prospective studies have been conducted evaluating its utility in long-term clinical outcomes. 

A recent study proposed a management algorithm tailored to correspond with BRAF status [36]. The study resulted in a 70% detection rate of BRAF on fine-needle aspiration in PTC patients and a positive predictive value of 100%. BRAF positive PTC patients were also more likely to have central lymph node metastases (47% versus 19%, *P* = 0.003). The study showed that BRAF positive patients were more likely to require cervical reoperations than BRAF negative patients (10% versus 3%, *P* = 0.04) [36]. The authors' further demonstrated that preoperative BRAF status could have beneficially altered the initial management in 24% of PTC. Based on the data, the authors' proposed algorithm entails routine ultrasound and lymph node mapping, cytologic BRAF testing of suspicious thyroid nodules and lymph nodes, and a total thyroidectomy for BRAF positive tumors [36]. Although the results demonstrated that BRAF positive patients were more likely to have central lymph node metastases, the authors stop short of recommending PCND in BRAF positive patients but rather recommend the selective approach as outlined in the ATA guidelines [1].

## 6. Conclusion

The debate surrounding the extent of initial thyroid surgery, the benefit of prophylactic neck dissection, and the need for radioactive iodine ablation persists and is likely to endure. The excellent prognosis of papillary thyroid cancer precludes recruiting the extensive number of patients required to conduct a meaningful prospective, randomized clinical trial focused on addressing the controversies of appropriate management. In its revised management guidelines, the ATA attempts to provide management guidelines based on risk stratification. Although this serves as the best current strategy, the divergence in expert opinion will continue to perpetuate the debate. The controversies regarding the extent of surgery and indications for RAI ablation will likely be addressed by future scientific breakthroughs in molecular prognostic markers that will help select individualized management.
